# Perineal recurrence of prostate cancer post-brachytherapy

**DOI:** 10.1259/bjrcr.20180104

**Published:** 2019-04-29

**Authors:** Sian Cooper, Toby Pillinger, Imtiaz Ahmed, Konrad Wolfe, Sidath Liyanage

**Affiliations:** 1 Southend University Hospital NHS Foundation Trust,; 2 Kings College Hospital NHS Foundation Trust,

## Abstract

We present a rare case of perineal recurrence of prostate cancer post low dose rate brachytherapy. Increased levels of prostate-specific antigen were recorded 12 years post brachytherapy. Pelvic CT and MRI visualized a nodular lesion in the perineum, and positron emission tomography demonstrated choline-avidity. Ultrasound-guided biopsy of the nodule was performed, yielding histology consistent with prostatic adenocarcinoma. Metastatic prostatic seeding to the perineum is a rare complication of brachytherapy. We discuss the putative mechanism, approach to diagnosis, and management.

## Clinical Presentation

**Figure 1.**
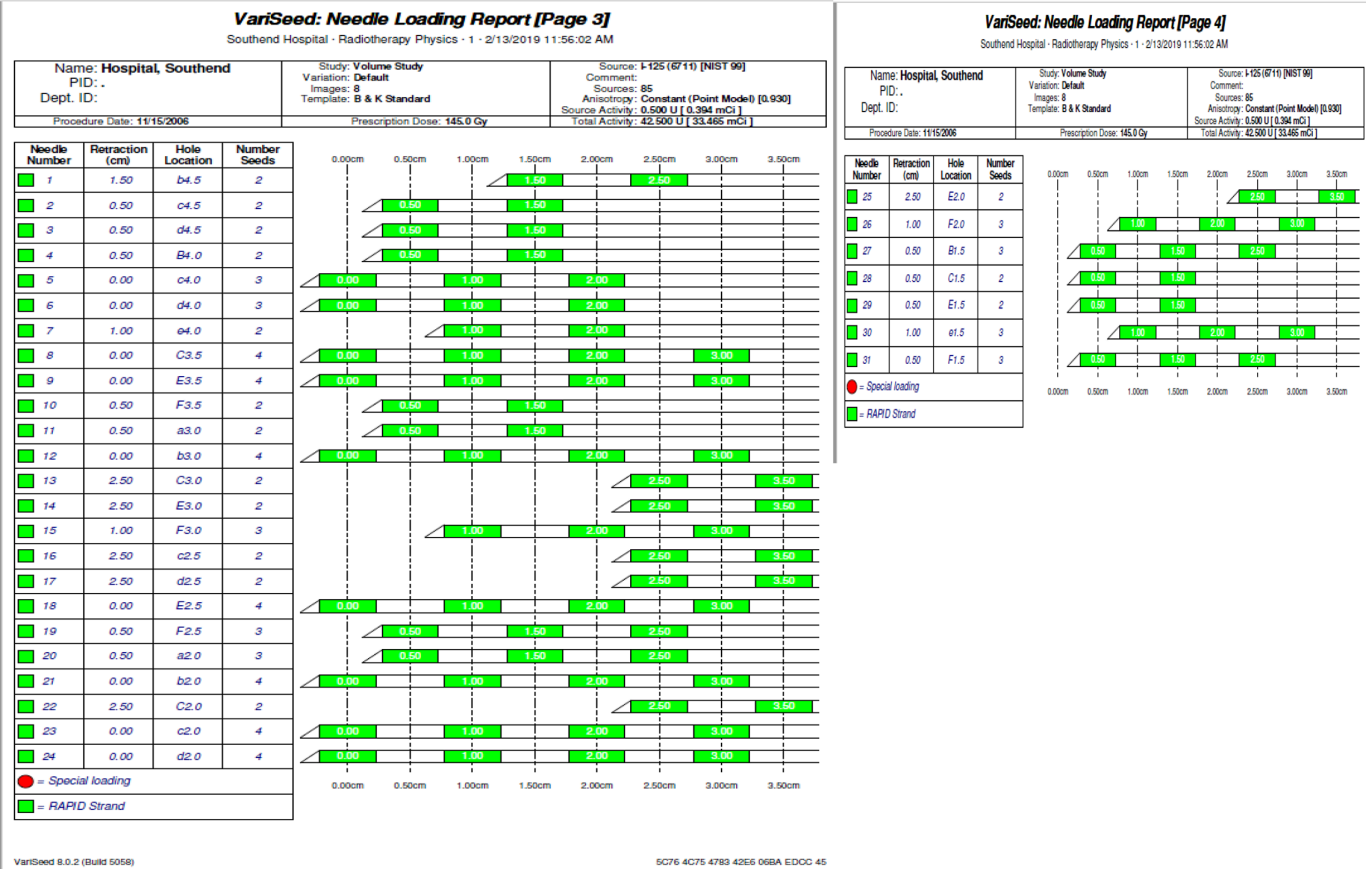
Seed implantation detail

**Figure 2.**
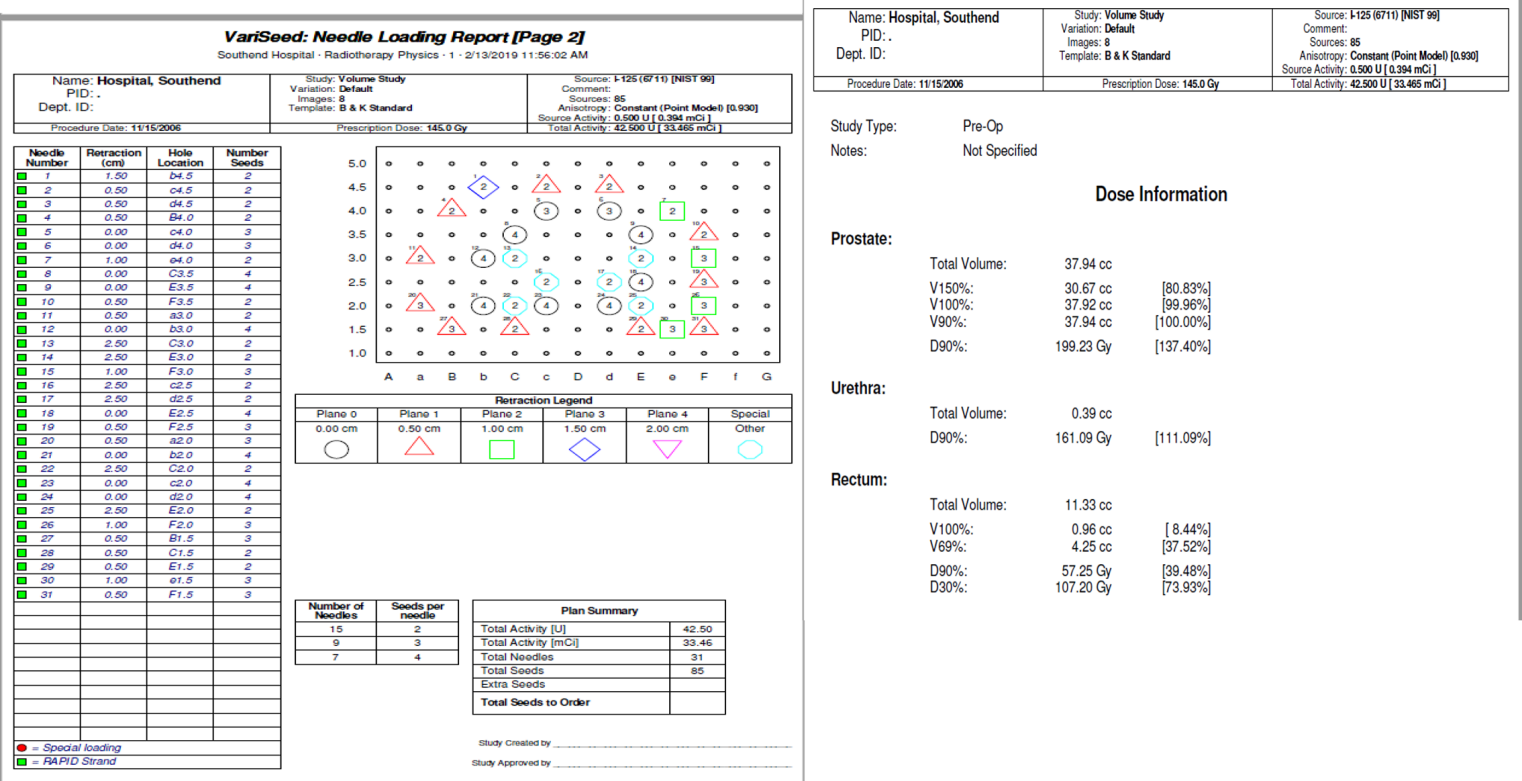
LDR dosimetry LDR, low dose rate.

**Figure 3.**
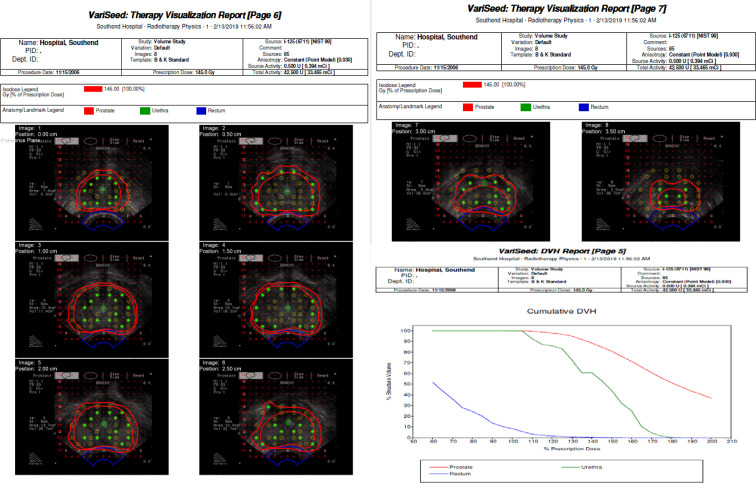
Planning imaging and DVH. DVH, dose volume histogram.

A 59-year-old male was diagnosed in 2006 with prostatic carcinoma. Initial prostatic specific antigen (PSA) titre was 10.3 µg l^−1^, histology demonstrated a Gleason score of 3 + 4 = 7, and clinical staging was recorded as T2N0M0. Past medical history was significant for diverticulosis and psoriatic arthritis. Regular medications were methotrexate, tamsulosin, and celecoxib and there were no known drug allergies. He underwent low dose rate (LDR) brachytherapy in November 2006 with no immediate complications. A total of 85 seeds were placed using 31 needles, and the prostate volume was 38.7 ml. Stranded seeds were manually loaded, with the required number cut. Figure 1 illustrates seed implantation detail. Iodine 125 was used. The dosimetric information, including planning imaging is shown in Figures 2 and 3. The following dosimetry was achieved: prostate V90% (>98%)=100.00%, prostate V150% (<85%)=80.83%, urethra D90% (<140%)=111.09% and rectum V100% (<2.00 cc)=0.96 cc. The procedure documentation reported no immediate complications. Estimated time of procedure was 30 min. There was no post-implant CT scan performed. The PSA nadir was established thereafter at 2.0 µg l^−1^. The immunoassay was Roche until February 2017, and Beckman was used thereafter.

He was subsequently monitored with serial PSA measurements as per NICE guidelines.^1^ In May 2016, annual screening recorded an acute rise in PSA titre to 4.99 µg l^−1^ in the absence of new urinary or gastrointestinal symptomatology. The PSA titre increased further to 6.47 µg l^−1^ 3 months later. He was investigated for disease recurrence. The PSA trajectory is outlined in Table 1.

**Table 1.** PSA trajectory

	**PSA**	**Date**	**PSA**
26 May 2006	10.3	06 August 2014	1.46
17 July 2006	11.2	20 July 2015	2.89
Brachytherapy		01 February 2016	4.99
24 January 2007	4.3	04 May 2016	4.73
17 May 2017	2.0	15 August 2016	6.47
30 August 2007	2.6	18 November 2016	8.04
03 December 2007	2.2	15 February 2017	9.76
28 May 2008	1.5	13 June 2017	10.10
01 December 2008	0.9	11 September 2017	11.05
28 May 2009	0.4	03 January 2018	26.12
04 January 2010	0.4	13 February 2018	18.99
07 January 2011	0.3	09 April 2018	17.67
04 January 2012	0.6	14 August 2018	0.02
07 January 2013	0.86	17 December 2018	<0.01
06 January 2014	1.6		

PSA, prostatic specific antigen.

Pleasenote Roche was used until 15.2.17, and Beckman thereafter.

## Investigations/Imaging findings

MRI of the prostate was performed including dynamic contrast-enhanced (DCE) imaging, which failed to identify tumour for targeted biopsy. Specifically, the prostate gland measured 4.9 × 2.7 × 3.3 cm in size (volume: 22.7 ml). Multiple brachytherapy seeds were visualised. The prostate gland demonstrated diffusely intermediate/low signal with no convincing areas of diffusion restriction (Prostate Imaging Reporting And Data System 2/5). On DCE, there were no areas of focal asymmetric pathological enhancement. The prostatic capsule and periprostatic fat planes were clear of metastatic spread. The seminal vesicles were atrophic. No significant pelvic lymphadenopathy was noted. There was no evidence metastasis in adjacent bones.

**Figure 4.**
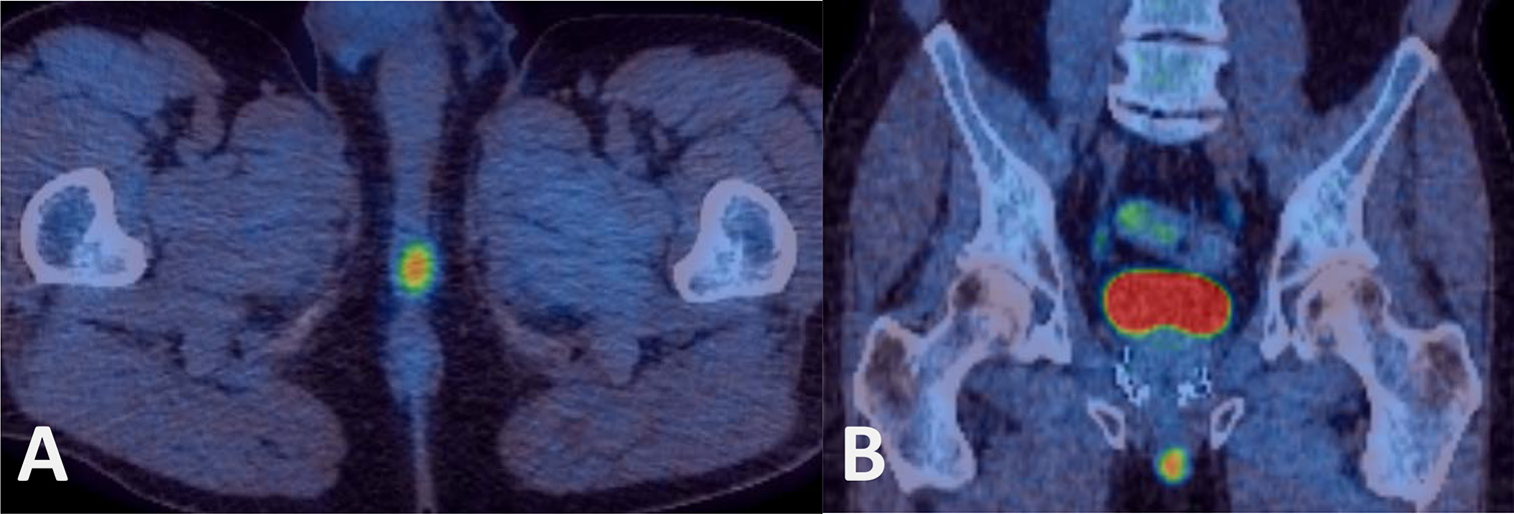
^18^F-choline PET-CT scan in the (A) axial and (B) coronal plane demonstrates a choline avid nodule (SUVmax = 8.8) at the base of the penis in the perineum. Brachytherapy seeds are present in the prostate gland in (B). PET, positron emission tomography; SUV_max_, maximum standardized uptake value.

**Figure 5.**
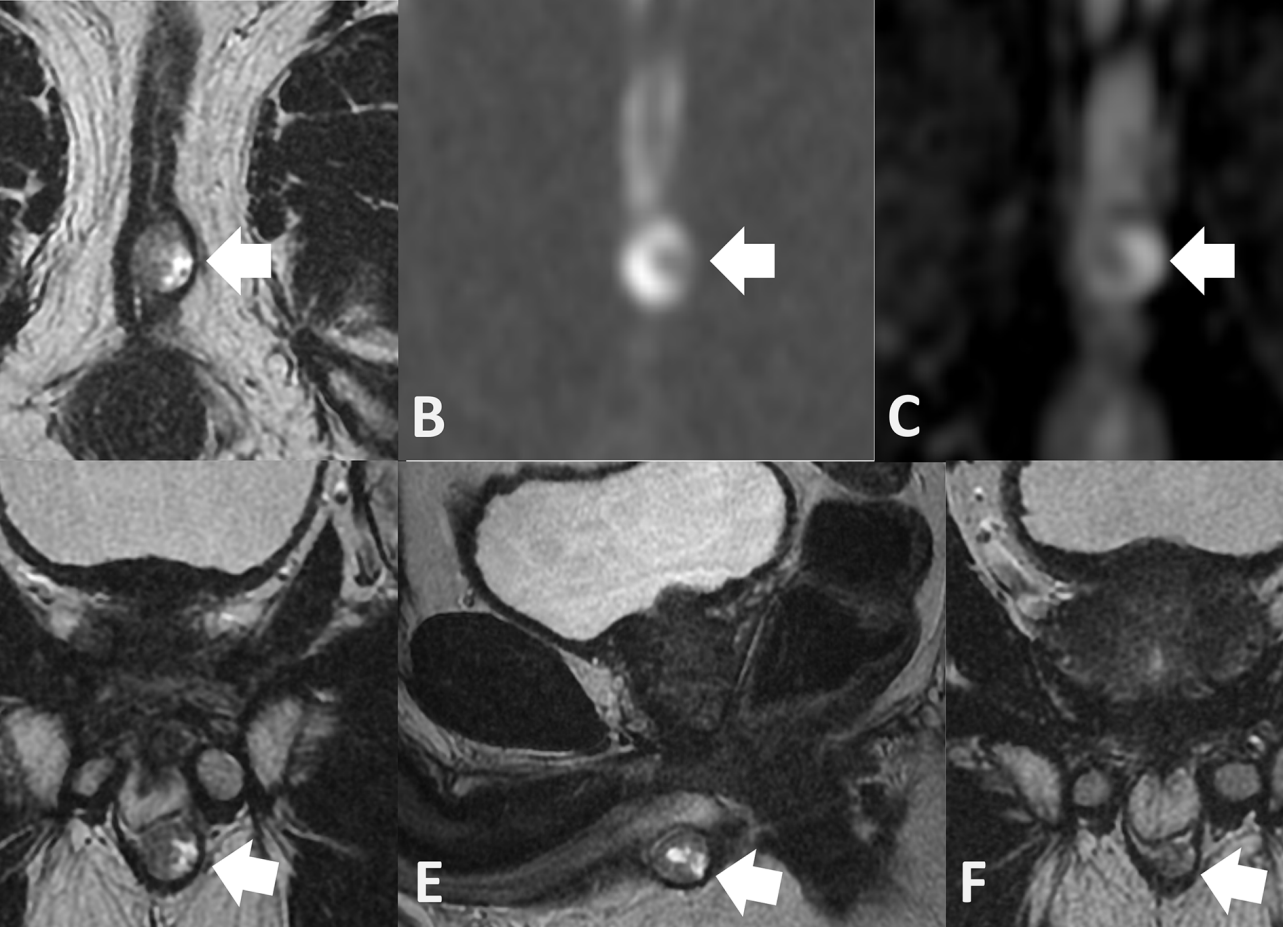
Axial *T*_2_ weigthed (A) DWI (B) and ADC map (C) shows a 15 mm mixed SI lesion (arrow) which is of high SI on the b-1400 diffusion scan and corresponding low SI on the ADC map indicating diffusion restriction. The lesion lies juxtaposed to the penile bulb and deep to the bulbocavernosus muscle on the coronal (D) and sagittal (E) scans. In retrospect, the lesion was present on the coronal *T*_2_ weighted MRI (F) performed 15 months earlier and is smaller (arrow). ADC, apparent diffusion coefficient; DWI, diffusion weighted imaging; SI, signal intensity.

Despite negative MRI findings, persistently elevated PSA indicated biochemical failure, and in February 2017 prostatic biopsies were taken. Histology demonstrated only radiation-induced atypia. [^11^C]-choline positron emission tomography (PET) was performed in October 2017 (Figure 4). This revealed a choline-avid nodule in the base of the penis, within the perineum. The area corresponded to an area of soft tissue nodularity on pelvic CT, and a repeat MRI of the pelvis 2 months later was suspicious for recurrence of disease at the bulb of the penis (Figure 5).

**Figure 6.**
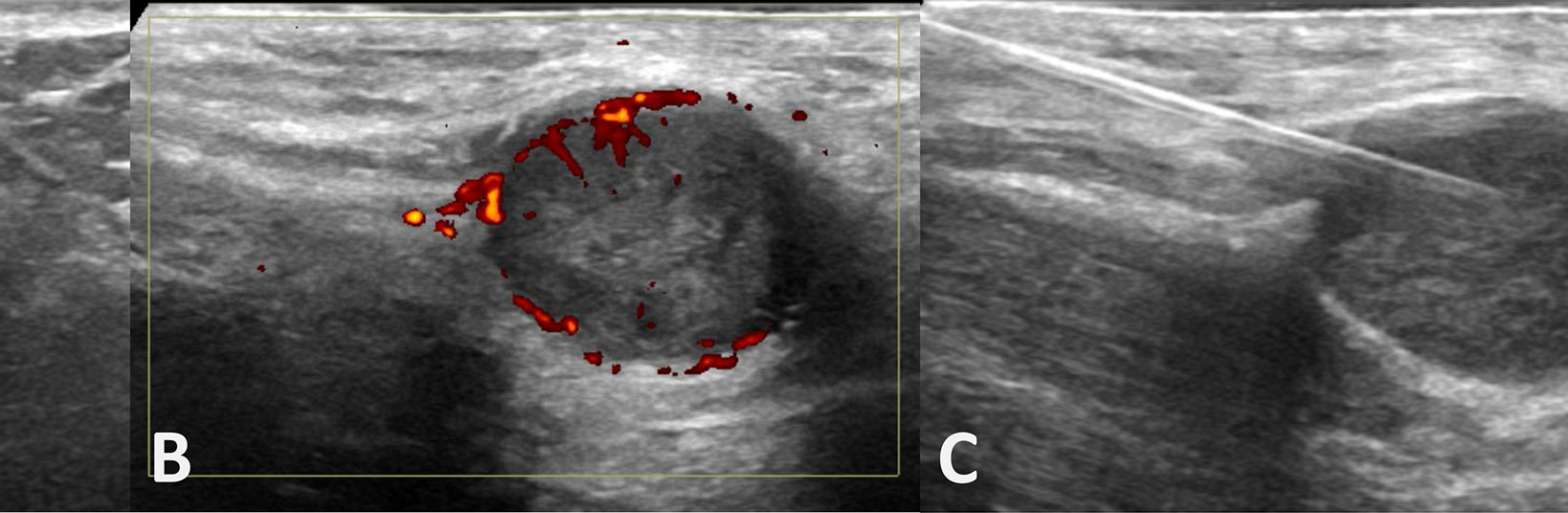
(A) Grayscale ultrasound shows a hypoechoic lesion. (B) There was posterior acoustic enhancement and peripheral power colour doppler flow. (C) Fine needle aspiration cytology of the lesion was performed under ultrasound guidance.

Ultrasound revealed a well-defined hypoechoic lesion at the base of the penis with posterior acoustic enhancement and some peripheral colour flow, correlating with the MRI findings (Figure 6).

**Figure 7.**
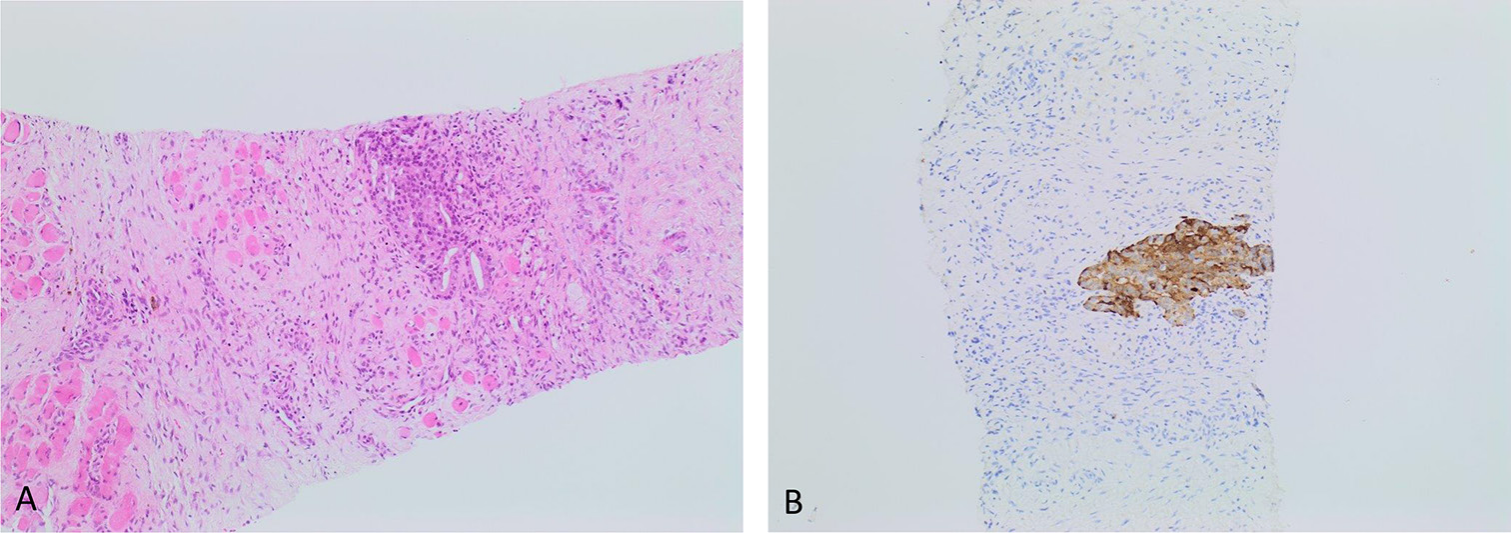
(A) Histology following fine needle aspiration cytology. (B) Histology with PSA stain.

Biopsy of the lesion yielded histology consistent with recurrent prostatic carcinoma involving the base of the penis (Figure 7A). To confirm the diagnosis, a Tru-Cut biopsy was performed. This revealed moderately differentiated adenocarcinoma, Gleason 4 + 4 = 8 (Figure 7B). CT of the chest abdomen and pelvis and bone scintigraphy confirmed absence of any further extra prostatic recurrence.

## Treatment

Androgen suppression (Leuprorelin) was commenced and he was referred for consideration of Cyberknife therapy in light of oligometastatic disease. He was accepted for salvage treatment, however, on repeat MRI and clinical review at the referral centre, the nodule had become too small to accurately place fiducials for Cyberknife. At time of writing, he continues on Leuprorelin therapy with ongoing clincial surveillance.

## Discussion

Perineal recurrence of prostate cancer is a rare complication of LDR brachytherapy, with only two previous cases described in the literature.^2,3^ 6 years had elapsed between brachytherapy and perineal relapse in one case^3^ and 4 years in the second.^2^ Our case describes a patient who relapsed 10 years after initial treatment, with no other surgical interventions in the interim.

The postulated mechanism of action is seeding of malignant cells by the brachytherapy needles. A previous review of 502 patients with prostate cancer who underwent perineal biopsy demonstrated that five patients (1%) developed perineal seeding.^4^ It appears to herald a poor prognosis: a subsequent case series by Moul and colleagues demonstrated that 100% of patients identified with seeding developed metastatic disease within 16 months, with a median survival of 36 months.^4^ Risk factors conferring increased chance of seeding were reported as large tumour volume and the use of Tru-Cut biopsy.^5^

Beyond perineal seeding, there is one reported case in the literature of bladder recurrence of prostate cancer post-brachytherapy presenting with biochemical failure.^6^ In this case, review of the CT scan obtained for dosimetry at the time of brachytherapy demonstrated a lone catheter protruding through the bladder wall at the site of eventual recurrence.

In terms of management of the recurrent nodule, options depend on the patient’s performance status, comorbidiy and preferences. In the literature, one patient was treated with volumetric arc radiotherapy to the localized recurrence.^3^ After multidisciplinary team discussion, our patient was commenced on luteinising hormone releasing hormone and referred for consideration of cyberknife ablation. The patient’s anatomy was not amenable to surgical resection.

With increasing incidence rates of prostate cancer diagnosis^7^ and subsequent treatment, the risk of iatrogenic complications such as that described in this case report will similarly rise. Moreover, improved survivorship of prostate cancer will increase rates of late complications of treatment.^8^ Thus, clinicians should consider seeding to the perineum in patients with biochemical failure who have previously undergone bracytherapy. Future studies are required to determine whether prompt diagnosis and expedient intervention in these patients improves prognosis.

## Learning points

1. The case presented demonstrates a rare late complication of prostate brachytherapy, identified by biochemical failure and subsequent imaging.
2. Little is known about the risk factors of perineal seeding, with only two previous similar cases reported in the literature.
3. Biochemical failure should alert the clinician to the possibility of seeding post brachytherapy.

## References

[1] NICE Prostate cancer: diagnosis and management Clinical guideline [CG175] [Internet]. 2014 Available from: https://www.nice.org.uk/Guidance/CG175.

[2] Teh BS, Chou CC, Schwartz MR, Mai WY, Carpenter LS, Butler EB Perineal prostatic cancer seeding following Radioactive seed brachytherapy. J Urol 2001; 166: 212. doi: 10.1016/S0022-5347(05)66116-2 11435863

[3] Eppinga W, Vijverberg P, Moerland R, Brand E, Zyp JvanderVvan, Noteboom J, et al Perineal recurrence of prostate cancer six years after trans-perineal brachytherapy. Jcb 2014; 4: 386–8. doi: 10.5114/jcb.2014.46753 PMC4300357 25834583

[4] Moul JW, Miles BJ, Skoog SJ, McLeod DG Risk factors for perineal seeding of prostate cancer after needle biopsy. J Urol 1989; 142: 86–8. doi: 10.1016/S0022-5347(17)38668-8 2733112

[5] Moul JW, Bauer JJ, Srivastava S, Colon E, Ho CK, Sesterhenn IA, et al Perineal seeding of prostate cancer as the only evidence of clinical recurrence 14 years after needle biopsy and radical prostatectomy: molecular correlation. Urology 1998; 51: 158–60. doi: 10.1016/S0090-4295(97)00487-1 9457312

[6] Raleigh DR, Hsu I-C, Braunstein S, Chang AJ, Simko JP, Roach M Bladder wall recurrence of prostate cancer after high-dose-rate brachytherapy. Brachytherapy 2015; 14: 185–8. doi: 10.1016/j.brachy.2014.11.008 25533416

[7] Cancer Registration Office of National Statistics [Internet] Cancer registration statistics, England Statistical bulletins. 2016 Available from: https://www.ons.gov.uk/peoplepopulationandcommunity/healthandsocialcare/conditionsanddiseases/bulletins/cancerregistrationstatisticsengland/previousReleases.

[8] CRUK Prostate Cancer Survival Statistics [Internet]. 2014 Available from: https://www.cancerresearchuk.org/health-professional/cancer-statistics/statistics-by-cancer-type/prostate-cancer/survival#ref-2.

